# A personalized medicine target: heart failure in women

**Published:** 2011-08-25

**Authors:** C Ginghin̆, CD Botezatu, M Serban, R Jurcuţ

**Keywords:** heart failure, women, ejection fraction, cardiomiopathy

## Abstract

It is increasingly known that gender differences affect disease presentation, clinical pathways, diagnostic yield and prognosis of patients with cardiovascular disorders. There are novel insights regarding heart failure that provide a platform for personalized medicine. This is a review of the existent data about heart failure in women, a neglected topic that has gained considerable interest in the past years.

Heart failure in women differs in many aspects from that of men. Part of the difference is attributable to age, ventricular function and cause of heart failure, with women being generally older at heart failure onset, more often without left ventricular systolic dysfunction and less often having heart failure due to ischaemic heart disease, in comparison with men. Elucidation of the genetic and pathophysiological basis of sex differences, together with clinical trials designed to study the impact of treatments in women, could lead to sex based heart failure management.

## Introduction

If until recently, epidemiological and clinical data regarding cardiovascular health were extrapolated to the entire population, recently published studies have shown that there are obvious, sex related differences, in terms of cardiovascular disease in general and heart failure in particular. The idea of a personalized approach of this pathology came into light with the evidence of different gene profiles in female and male. Autosomal genes (GATAD1, SCLA12, PDE6B) involved in the metabolism of cyclic nucleotides, glucose transport and neurohumoral stimulation were identified in women with heart failure, unlike the male population with heart failure, where the genes responsible for arrhythmias, self immunity and cellular homeostasis (KCNK1, CD24, PLEKHA8) were overexpressed [1] (Table 1). It is believed that gender-specific differences in gene expression may explain the physiopathological and clinical differences between sexes regarding heart failure. In this context, personalized medicine gained an increasing interest, the target being the understanding of the impact of gender differences, establishing a strictly individualized disease approach and obtaining the leads for treatment optimization.

**Table 1:** Heart failure: gender–specific differences in gene expression (modified after [1,2]).

WOMEN	MEN
Regulation in gene expression of adrenergic and angiotensin signaling (GATAD1)	Regulation in gene expression of potassium channel (KCNK 1)
Regulation in gene expression of glucose transporter (SLCS2A12)	Autosomal gene CD24, that through its expression on leucocytes may have an important function in immunological processes
Regulation in gene expression of phosphodiesterase (PDE6B)	Regulation in gene expression of cellular homeostasis (PLEKHA 8)

Although more than half of the patients diagnosed and hospitalized for heart failure are women, they are underrepresented in most of the clinical studies. Even in recent trials, only 20% of patients with heart failure are women. This explains the limited analysis of sex related differences in cardiovascular pathology [3].

## Etiology of heart failure

There are gender differences regarding the age at heart failure onset, as well as heart failure etiology and risk factors in patients with heart failure [4]. Unlike men with heart failure, in whom the ischemic etiology prevails, the main risk factors in women are arterial hypertension, valvular heart disease and atrial fibrillation [5] (Table 2).

Women develop heart failure at higher age and this is why they cumulate more risk factors [6]. Many studies have found arterial hypertension to be the most important risk factor for heart failure in women. The significant prevalence in women over 55 years old has been attributed to higher systolic blood pressure after menopause. A strong correlation between hypertension and heart failure has also been observed in young afro-American women with uncontrolled blood pressure.

**Table 2:** Etiologic spectrum of heart failure in women (according to 5,12).

MAIN CAUSES	PARTICULAR FORMS
Arterial hypertension Diabetes mellitus Obesity Valvular heart disease Vulnerability to ischemia	X–linked cardiomyopathy Peripartum cardiomyopathy Tako–Tsubo cardiomyopathy Toxic cardiomyopathy (ethanol, antracicline)

*Diabetes mellitus* is associated with an increased risk of heart failure in female patients, this being proved since the Framingham study: the incidence of heart failure was 4–5 times higher in diabetic women compared to non–diabetic women and twice higher than in diabetic male subjects. The probability for diabetic patients to develop heart failure is strongly linked to the glycemic control. A study made on more than 48.000 diabetic patients showed a probability of 4.2/1000/year at a HbA1c value of less than 7% and a probability of 9.2/1000/year at a HbA1c value of more than 10% [7]. The endothelial dysfunction is a characteristic of diabetes, and endothelium–derived substances may have profound effects on myocardial structure and function. For example, both endothelin and angiotensin II cause myocardial hypertrophy and increased interstitial connective tissue, which can explain the higher left ventricle mass in patients who associate diabetes and hypertension, compared to nondiabetic hypertensive patients. Therefore, it is not surprising that diabetes is one of the major factors, along with hypertension and coronary heart disease, associated with heart failure with normal systolic function, form frequently observed in women.

*Obesity*, an integral component of the metabolic syndrome and type 2 diabetes,  predisposes to the toxic effects of fatty acids on the myocardium and the additional detrimental effects of cytokines and angiotensin II released by adipose tissue. Therefore, obesity is a risk factor for heart failure for both men and women. In the Framingham study, the risk of heart failure was twice higher for the obese persons compared to subjects with normal body weight [8].

*Valvular heart disease* continues to be a cause of heart failure in women. According to the data published by Redberg et al., 70% of the patients diagnosed with mitral or aortic valve disease are women [9]. Rheumatic mitral stenosis due to rheumatic fever in childhood used to be a major cause of valve disease in female. This still is a major contributor of heart failure in some part of the underdeveloped world. In developed countries, a more common cause of heart failure is the degenerative valvular disease with aortic stenosis, this being the classic example.

Although the incidence of coronary heart disease is lower in women, the increased vulnerability to ischemia is notable [10]. The studies have shown that, regardless of the type of treatment (conservative/myocardial revascularization), women who suffered a myocardial infarction are more predisposed to develop heart failure [11].

Several types of *cardiomyopathy* are more frequently linked to the female sex. It has been consistently seen in literature that women are very vulnerable to an increased level of catecholamines, 90% of stress *induced cardiomyopathies* (Tako–Tsubo) being registered in women. Differences between men and women were also described regarding *cardiotoxicity*. At similar doses of chemotherapy (anthracycline class) or ethanol, women have a higher risk of developing heart failure.

*Peripartum cardiomyopathy* is a particular case of heart failure with an etiology that is not completely known, with nonspecific diagnostic criteria and reserved prognosis. The incidence varies according to different studies and has been estimated to range from 1:2300 to 1:4000 pregnancies in Western countries. A geographical predilection is found in Haiti, with an estimated incidence of 1:299. Advanced maternal age, multiparity, multifetal pregnancy, preeclampsia and gestational hypertension are mentioned among the risk factors. Recently, the overexpression of 16–kDa prolactin was suggested to be a possible physiopathological mechanism for peripartum cardiomyopathy [12]. This proposed pathway emphasizes a coincidence of unbalanced oxidative stress, activation of the protease cathepsin D, that cleaves the nursing hormone prolactin into an angiostatic and proapoptotic 16–kDa form, which seems to drive the disease by systemically affecting the endothelium, as well as the cardiac vasculature and cardiomyocyte function. Consistent with this idea, blockade of prolactin by bromocriptine, a dopamine D2–receptor agonist, prevented the onset of disease in an experimental model of peripartum cardiomyopathy and appeared successful in small pilot trials. In the presence of risk factors and clinical suspicion in young women at the end of pregnancy or in the first 5 months postpartum, the diagnosis of  peripartum cardiomyopathy, based on exclusion criteria, is established by ultrasound investigation once the systolic dysfunction appears (the decrease of the ejection fraction under 45% and/or fractional shortening under 30%). The prognosis is reserved because of the limited recovery (23–32% of all cases) and the high mortality rate (15% of all cases) [12].

When speaking about *X–linked cardiomyopathy* in women, the slow progression and the late onset are remarkable features, females being heterozygote for the mutant allele. At the opposite pole, men with this condition become symptomatic since childhood.

From the associated pathology point of view, *thyroid dysfunction* is more frequent in women with acute heart failure, while in men, obstructive pulmonary disease, peripheral vascular disease, and renal failure prevail. 

## Physiopathological particularities

There is no opinion agreement regarding the existence of physiopathological particularities linked to sex because it is difficult to exclude the variables that could be responsible for those particular elements.

Starting from the observation that women have a higher incidence of heart failure with preserved ejection fraction but with reduced diastolic compliance, the issue of sex–related variability of cardiac function came into light. The answer was given by a study that analyzed the diastolic compliance in healthy teenagers. For female gender, higher body mass index, ventricular rate, total peripheral stiffness and relative left ventricle walls thickness were observed. Lower values were registered for the E/A ratio, suggesting that women have a higher probability to develop diastolic dysfunction even independent of the additional risk factors. The reason for those differences in filling and relaxation is not fully known. Although E/A ratio can depend on ventricular rate, blood pressure, geometry and left ventricular systolic function, the multivariate analysis showed that sex had a significant influence on ventricular filling [13].

Studies on adult patients showed, as a particular element in women, a higher capacity of the left ventricle to adapt to overload through concentric hypertrophy, unlike men who had a predisposition to ventricular dilation and contractile dysfunction [14,15]. Although the cause of this different response is not entirely understood, a reasonable explanation is offered by the sex–dependent variability of vascular and ventricular stiffness. There is a significant increase of vascular stiffness in women, with age. In order to maintain the optimal ventriculoarterial interaction, the ventricle develops increasing elasticity, both systolic and diastolic, which has the advantage of preserving the ejection fraction and the disadvantage of altering the relaxation. It remains a dilemma what the factors that determine the increase of arterial stiffness in women are. Among the proposed mechanisms, endothelium dysfunction is a notable factor, frequent in women, as well as the differences in protein structure and in neurohumoral signal [16].

## Clinical presentation

The severity of symptoms is one clinical particularity of heart failure in women. The MONICA study (Multinational MONItoring of trends and determinants in CArdiovascular disease) showed that, in similar contractile parameters, women are more symptomatic than men [17]. The prevalence and the increased severity of symptoms were attributed on one hand to the lower level of pain perception, and on the other hand to depression. The neurological disorders like loss of memory and depression were correlated to a significant hippocampus reduction in women with heart failure, compared to the control group and to male subjects with heart failure. Because women frequently have the tendency to empathize, the objective evaluation through clinical score systems is more difficult to achieve and the obtained data have usually reduced accuracy.

## Paraclinical data

Both quantitative and qualitative analyses of cardiac function have been rarely evaluated in female subjects. It is possible to have some differences between sexes regarding the paraclinic data, but because of the less frequently used diagnostic investigations, so far they have not been assessed [18,19].

*B–type natriuretic peptide*, a biomarker used for risk stratification in patients with heart failure, registers higher values in normal females [20]. Therefore, the use of a different cutoff for the diagnosis and management of heart failure for the two sexes was suggested. The BASEL study (B–type natriuretic peptide for Acute Shortness of Breath Evaluation), that had as main purpose the  analysis of the prognostic role of BZ–type natriuretic peptide (BNP) in patients with acute dyspnea, showed that a higher level of BNP is a stronger predictor of death in women. The risk was 5.1 times higher in women with BNP> 500 pg/ml than in those with BNP values below 500 pg/ml, and for similar BNP values in men, the difference in risk was of only 1.8 [21]. However, further studies that enroll a sufficient number of female patients are needed to analyze the distribution of heart failure biomarkers and its prognostic significance.

The *electrocardiogram* is among the first tests available for the evaluation of postmenopausal women. Repolarization abnormalities are prognostic predictors of heart failure and all–cause mortality, as significant as the presence of an old myocardial infarction on ECG; therefore, this should not be overlooked. The presence of repolarization abnormalities, particularly wide QRS/T angle, may make a substantial difference in the number of new heart failure events and may warrant consideration of possibly intensified prevention efforts. A wide angle, expression of structural and functional myocardial abnormalities, increases the risk of heart failure by three times. Two other dominant repolarization variables, ST depression in V5, tall T wave in V1 lead, and QRS non–dipolar voltage were associated with a 2–fold increase in the risk of incident heart failure having the same prognostic value as a myocardial infarction [22].

Through the analysis of heart frequency variability, the *ECG Holter monitoring* may bring information with prognostic value. There are studies that showed a strong correlation between a high variability of ventricular rate and heart failure [22].

The effort capacity, evaluated through the 6-minute walk test is significantly higher in men with heart failure, compared to women with similar systolic dysfunction. In the First study, the effort tolerance and the mean pulmonary capillary wedge pressure proved to be independent predictors for survival in patients with heart failure [23].

From the *echocardiography* point of view, the preservation of left ventricular systolic function is characteristic in women with heart failure. This was also the conclusion of the Framingham study, which showed that women represent 65% of patients with diastolic heart failure and only 25% of those with systolic dysfunction.

## Diagnosis algorithm of heart failure in women

Studies show that the accuracy of heart failure diagnosis by clinical means alone is often inadequate, particularly in women. The diagnosis is established late in most of the female patients, clinical manifestations being attributed to other causes. Women were less likely to be hospitalized in a cardiology department and therefore have less chances of accurate assessment of cardiac function. In these circumstances, it is not surprising that heart failure in women is treated to a lesser extent according to present recommendations [24].

**Figure 1:**
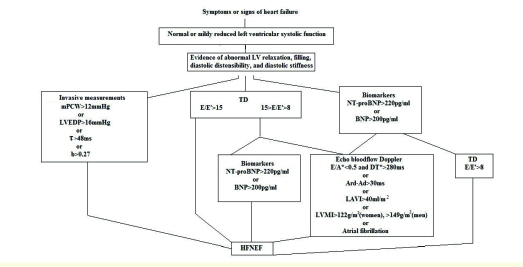
Diagnostic flowchart on How to diagnose HFNEF in a patient suspected of HFNEF (according to [26]). LVEDVI, left ventricular end–diastolic volume index; mPCW, mean pulmonary capillary wedge pressure; LVEDP, left ventricular end–diastolic pressure; t, time constant of left ventricular relaxation; b, constant of left ventricular chamber stiffness; TD, tissue Doppler; E, early mitral valve flow velocity; E0, early TD lengthening velocity; DT, deceleration time; LVMI, left ventricular mass index; LAVI, left atrial volume index; Ard, duration of reverse pulmonary vein atrial systole flow; Ad, duration of mitral valve atrial wave flow, >50 years old.

In the context of reduced BNP dynamics, preserved ejection fraction and normal ventricular dimensions, heart failure is frequently underdiagnosed in women. Because of the high diastolic heart failure prevalence, it is necessary to know the three diagnostic elements: signs and symptoms of heart failure, normal left ventricle function or marginally abnormal (FEVS>50%), proofs of abnormal left ventricular relaxation [25]. The authors of the European Consensus regarding the diagnostic of heart failure with preserved ejection fraction [26], published in 2007, proposed a comprehensive algorithm for the clinical approach of this entity (Figure 1).

## Treatment

The therapy particularities in women were for a long time underestimated due to their absence from the big therapy trials. When women were included in studies, their number was significantly lower compared to men, and therefore the conclusions were incorrect, e.g. the modest benefit of angiotensin–converting enzyme inhibitors in female patients in SOLVD trial. This is one of the explanations for the suboptimal treatment in women with heart failure, regardless of age [27]. More men are treated with angiotensin–converting enzyme inhibitors, in part reflecting the high proportion of men with systolic dysfunction. A multicentric European study showed that fewer women with evidence of left ventricular systolic dysfunction were treated with drugs that have a documented impact on survival (angiotensin–converting enzyme inhibitors, β–blockers and spironolactone), whereas they receive more often cardiac glycosides and loop diuretics [28].

An inverse correlation between plasma levels of omega–3 polyunsaturated fatty acids and incidence of heart failure was detected exclusively in women [29]. Moderate consumption of fish (1–2 meals/week) and omega–3 fatty acids was associated with a lower rate of hospitalization and mortality in women with heart failure [30].

Knowing the protective effect of hormones, the treatment with estrogen and combination estrogen–progesterone derivatives has been proposed. However, hormonal therapy has not proved effective in preventing heart failure or increasing survival [31]. Starting from the premise of muscle loss and reduced exercise capacity, testosterone treatment has been tried. In clinical trials, the results were encouraging, proving to increase muscular strength, and exercise tolerance and to reduce insulin resistance in women with advanced heart failure [32]. 

In a retrospective analysis of the SOLVD trial, women with heart failure had an increased risk of thromboembolic events compared with men. Although women have experienced more thromboembolic events (especially pulmonary), antiplatelet therapy and oral anticoagulant has been less frequently used in women [33].

A sub–analysis of MADIT–CRT trial has revealed that women with heart failure have a particularly favorable response to cardiac resynchronization therapy with defibrillator function. Heart failure related events were reduced by 70% in women and only 35% in men. The same study showed a 72% reduction in all–cause mortality in women with heart failure who received resynchronization therapy with defibrillator function. An update of the 2008 ESC guidelines for the diagnosis and treatment of acute and chronic heart failure, and the 2007 ESC guidelines for cardiac and resynchronization therapy, sustain that substantial improvements in left ventricle size and function were greatest in patients with a QRS width of >150 ms, patients with left bundle, patients with non–ischaemic etiology, and in female patients [34], hence suggesting that a good accessibility of women to this therapy would be of great benefit in this population.

Another recent report described that heart failure medication was not prescribed to target dose as recommended in the guidelines. Here, physician gender also played a role, with female patients treated by male physicians being least likely to receive the full dosage, and female doctors being more likely to prescribe target doses of heart failure medication to female patients [35].

There are also gender differences regarding drug pharmacokinetics and pharmacodynamics. If the absorption, transport and volume of distribution did not differ significantly between women and men with heart failure, excretion and metabolism explains their different effects depending on gender. On average, glomerular filtration rate (GFR) is higher in men than in women. This difference is not gender–related; rather, it represents a body mass effect, in that GFR is directly proportional to weight and men typically outweigh women. In addition, women generally produce less creatinine than men, since they have less muscle mass, and muscle mass is the major determinant of creatinine generation. It follows that women have a lower normal range for serum creatinine values and that identical serum creatinine values in a man and a woman reflect considerably different GFRs. Circumstances such as these make serum creatinine a provisional indicator of renal function. Because GFR values are somewhat lower in women, a number of cardiovascular medications–including angiotensin–converting enzyme inhibitors, certain β blockers, several antiarrhythmics, and digoxin will systemically accumulate upon repeat dosing unless medication doses are reduced.

Supplementary, the metabolic rate of drugs through the cytochrome P450 is reduced in female patients. In this context, we need to assess the complications associated with the pharmacological treatment that are more frequently reported in women. These differences are especially evident in the risk of proarrhythmic responses of women to medications that prolong the QT interval. Women are at greater risk for torsade de pointes from antiarrhythmic medications due to longer baseline QT intervals, than men, and a greater degree of QT prolongation for the same drug levels compared with men. Despite careful dosing based on creatinine clearance, the incidence of torsade de pointes with dofetilide is higher in women than in men. Regardless, there are no gender–specific guidelines for the administration of these agents. Therefore, there is evidence that the commonly used indicators of risk for malignant arrhythmias may be less sensitive for women than for men. The question may then be raised whether separate criteria are needed for risk stratification and treatment of arrhythmias in men and women. The answer to this question is far from clear–largely due to the limited data. Furthermore, female–specific issues such as pregnancy, menopause, oral contraceptive use, and menstruation may independently influence drug metabolism and serve as confounders to the interpretation of gender differences in drug handling or effect [36].

With the discovery of prolactin effects on the cardiovascular system: the impairing of remodelation and vascular formation, the destruction of cardiac microvascularization, the interference with cardiomyocyte metabolism, the decrease of the ejection fraction and ventricular dilatation, appeared the premises of the new therapy approach. Based on experimental observations that prolactin blockade with bromocriptine prevented the onset of peripartum cardiomyopathy, a specific treatment for these diseases has been developed [12].

Females are also poorly represented in cardiac rehabilitation programs. The low participation is attributed to older age, reduced tolerance to effort and associated comorbidities. Studies have shown the existence of socio–familial differences, which could explain the low number of women who benefit from cardiac rehabilitation: women have more family and social duties, but more difficulties in terms of financial resources, transport, or family support. However, no sex–dependent differences in relation to the benefit of cardiac rehabilitation were demonstrated.

## Cautions

Pregnancy may lead to deterioration of heart failure due to the rise in blood volume and increase in cardiac output, as well as the substantial increase in extravascular fluid. The risk of pregnancy is considered greater than the risks linked to contraceptive use. It is recommended that women with heart failure discuss contraceptives and planned pregnancy with a physician in order to take an informed decision based on assessment of potential risks (class I recommendations, C level of evidence: ESC Guidelines for the diagnosis and treatment of acute and chronic heart failure 2008).

### Prognosis

Heart failure has become the most important public health problem in cardiovascular medicine. The incidence of heart failure has increased with 9% in women and 6% in men during the last 20 years [37]. Mortality at 5 years after diagnosis reached 75%, being surpassed only by the one registered in bronchopulmonary cancer.

The FIRST trial (Flolan Randomizen Survival Trial) showed a two times higher rate of survival for women with advanced heart failure compared to men. Female sex has favorable prognosis regarding all–cause mortality, cardiovascular related death and hospitalization for heart failure. Subgroup analysis suggests this finding is strongest among patients with heart failure with preserved ejection fraction and in patients with nonischemic heart failure. Better understanding of the biological and psychosocial factors that contribute to the survival advantage of women with heart failure may ultimately allow improved management of patients with this deadly syndrome.

### Conclusions

For a long period of time, women have been profoundly underrepresented in clinical trials, and the influence of sex on cardiovascular pathophysiology was neglected. Recent studies showed significant differences between men and women with heart failure. Although, more clinical trials are needed in this field, the current tendency towards a similar approach to all patients with heart failure has to be changed.
